# Low pre‐exercise muscle glycogen availability offsets the effect of post‐exercise cold water immersion in augmenting PGC‐1α gene expression

**DOI:** 10.14814/phy2.14082

**Published:** 2019-06-03

**Authors:** Robert Allan, Adam P. Sharples, Matthew Cocks, Barry Drust, John Dutton, Hannah F. Dugdale, Chris Mawhinney, Angela Clucas, Will Hawkins, James P. Morton, Warren Gregson

**Affiliations:** ^1^ Research Institute for Sport and Exercise Sciences Liverpool John Moores University Liverpool UK; ^2^ Division of Sport, Exercise and Nutritional Sciences University of Central Lancashire Preston UK; ^3^ Institute for Science & Technology in Medicine School of Medicine Keele University Staffordshire UK; ^4^ Norwich Medical School University of East Anglia Norwich UK; ^5^ Medical Research Council Functional Genomics Unit Department of Physiology, Anatomy and Genetics University of Oxford Oxford UK; ^6^ College of Sports Science and Technology Mahidol University Nakhon Pathom Thailand

**Keywords:** Carbohydrate, cooling, skeletal muscle, training adaptation

## Abstract

We assessed the effects of post‐exercise cold‐water immersion (CWI) in modulating PGC‐1α mRNA expression in response to exercise commenced with low muscle glycogen availability. In a randomized repeated‐measures design, nine recreationally active males completed an acute two‐legged high‐intensity cycling protocol (8 × 5 min at 82.5% peak power output) followed by 10 min of two‐legged post‐exercise CWI (8°C) or control conditions (CON). During each trial, one limb commenced exercise with low (LOW: <300 mmol·kg^−1^ dw) or very low (VLOW: <150 mmol·kg^−1^ dw) pre‐exercise glycogen concentration, achieved via completion of a one‐legged glycogen depletion protocol undertaken the evening prior. Exercise increased (*P* < 0.05) PGC‐1α mRNA at 3 h post‐exercise. Very low muscle glycogen attenuated the increase in PGC‐1α mRNA expression compared with the LOW limbs in both the control (CON VLOW ~3.6‐fold vs. CON LOW ~5.6‐fold: *P *=* *0.023, ES 1.22 Large) and CWI conditions (CWI VLOW ~2.4‐fold vs. CWI LOW ~8.0 fold: *P *=* *0.019, ES 1.43 Large). Furthermore, PGC‐1α mRNA expression in the CWI‐LOW trial was not significantly different to the CON LOW limb (*P *=* *0.281, ES 0.67 Moderate). Data demonstrate that the previously reported effects of post‐exercise CWI on PGC‐1α mRNA expression (as regulated systemically via β‐adrenergic mediated cell signaling) are offset in those conditions in which local stressors (i.e., high‐intensity exercise and low muscle glycogen availability) have already sufficiently activated the AMPK‐PGC‐1α signaling axis. Additionally, data suggest that commencing exercise with very low muscle glycogen availability attenuates PGC‐1α signaling.

## Introduction

It is well documented that regular endurance training induces an increase in skeletal muscle mitochondrial density (Holloszy 1967). At a molecular level, the mitochondrial adaptations induced by endurance training are largely regulated via transient increases in mRNA transcripts encoding mitochondrial proteins in response to each acute training session (Perry et al. 2010). Upon the onset of contraction, homeostatic perturbations within skeletal muscle (e.g., increased AMP/ATP ratio, Ca^2+^, reactive oxygen species (ROS), lactate, reduced glycogen availability, etc.) result in the activation of regulatory protein kinases that, in turn, activate downstream targets such as transcription factors or transcriptional coactivators (Ljubicic and Hood 2009). As a transcriptional coactivator, the peroxisome proliferator‐activated receptor coactivator (PGC‐1α), has been the focus of intense investigation during the last two decades and is repeatedly cited as the “master regulator of mitochondrial biogenesis” (Bartlett et al. 2012; Puigserver and Spiegelman 2003). The importance of PGC‐1α in regulating mitochondrial content and function is evident from rodent studies demonstrating that overexpression increases oxidative enzyme activity (Lin et al. 2002), improves insulin sensitivity (Benton et al. 2008), protects against sarcopenia (Wenz et al. 2009) and also improves exercise capacity (Calvo et al. 2008).

In relation to human skeletal muscle, multiple laboratories have examined the potential to augment the adaptive response to a given exercise stimulus through interventions that modulate and enhance the exercise‐induced activation of the PGC‐1α signaling axis. Consistent with the initial discovery that PGC‐1α was cold‐inducible in rodent skeletal muscle (Puigserver et al. 1998), we (Allan et al. 2017; Joo et al. 2016) and others (Ihsan et al. 2014, 2015) have demonstrated that both passive and post‐exercise cold‐water immersion (CWI) enhances the acute expression of PGC‐1α mRNA (Joo et al. 2016), an effect that is likely regulated systemically (via β‐adrenergic activation of local cell signaling pathways), as opposed to local cooling effects per se (Allan et al. 2017). In accordance with the acute effects of CWI, chronic application of the CWI stimulus in response to consecutive training sessions up‐regulates chronic markers of training adaptations such as lipid enzyme activity and oxidative enzyme protein content (Ihsan et al. 2015).

In addition to CWI, we (Bartlett et al. 2013; Impey et al. 2016) and others (Hulston et al. 2010; Psilander et al. 2013; Van Proeyen et al. 2011; Yeo et al. 2010) have also demonstrated a potent role of reduced muscle glycogen availability in enhancing the chronic adaptations to endurance training, an effect that is also associated with the augmented activation of the AMPK‐PGC‐1α signaling axis in response to an acute training session that is completed with reduced CHO availability before, during, and/or after exercise (Impey et al. 2018). This body of work is often communicated as the train‐low (smart): compete high paradigm surmising that carefully selected training sessions could be completed with reduced CHO availability so as to augment training adaptation, yet competition should always be commenced with high CHO availability so as to promote optimal performance. When taken together, such data raise the possibility that simultaneous application of post‐exercise CWI and reduced CHO availability may augment the cell signaling responses associated with the regulation of mitochondrial biogenesis, when compared with the application of either stimulus in isolation. However, given recent data highlighting the role of local muscle metabolic stress in modulating acute exercise‐induced cell signaling pathways (Fiorenza et al. 2018), it is suggested that the application of CWI (i.e., a systemic mediated stress) induces negligible regulatory effects on a muscle that has already been subjected to the extreme local metabolic challenge of both high‐intensity exercise and low muscle glycogen availability.

Accordingly, the aim of the present study was to assess the effects of post‐exercise CWI in modulating the regulation of PGC‐1α mRNA expression in muscles that have already completed the challenge of high‐intensity exercise and low muscle glycogen availability. Using a prior glycogen manipulation protocol, we adopted an experimental design where subjects completed an acute two‐legged high‐intensity cycling protocol with and without two‐legged post‐exercise CWI but where each limb commenced exercise with low (<300 mmol·kg^−1^ dw) or very low (<150 mmol·kg^−1^ dw) pre‐exercise glycogen concentration. In this way, we were able to obtain muscle biopsies from four limbs subjected to the same exercise stimulus but with differing local (i.e., pre‐exercise glycogen availability) and systemic stressors (i.e., CWI vs. non‐cooling conditions).

## Materials and Methods

### Participants

Nine recreationally active healthy males (age 22 ± 3 years; body mass 74.18 ± 7.88 kg; height 180.50 ± 6.60 cm; peak power output (PPO) 272 ± 256 W; mean ± SD) participated in this study. Participants were instructed to refrain from exercise, alcohol, and caffeine 48 h prior to the first depletion protocol, and not to stray from the prescribed meal plan or exercise within the 48 h preceding the experimental day. All procedures performed in the study were approved by the Ethics Committee of Liverpool John Moores University and in accordance with the 1964 Helsinki declaration and its later amendments.

### Preliminary testing

Prior to commencing the experimental trials the participants completed an incremental exercise test to fatigue for the determination of $\dot{V}O_{2}$ peak and PPO (as described in detail in Hawley and Noakes 1992). Results from this test were used to determine the Watts necessary for cycling at a proportion of PPO on subsequent test days. PPO was calculated using the equation below (Pedersen et al. 2008); where CB is the wattage of the last complete bout, FB is the fraction of the final bout completed, and 25 is the increment of 25W between each successive bout: PPO = CB + (FB × 25). Further preliminary visits encompassed familiarization to the glycogen depletion protocols to be completed prior to the experimental day.

### Experimental design

**Figure 1:**
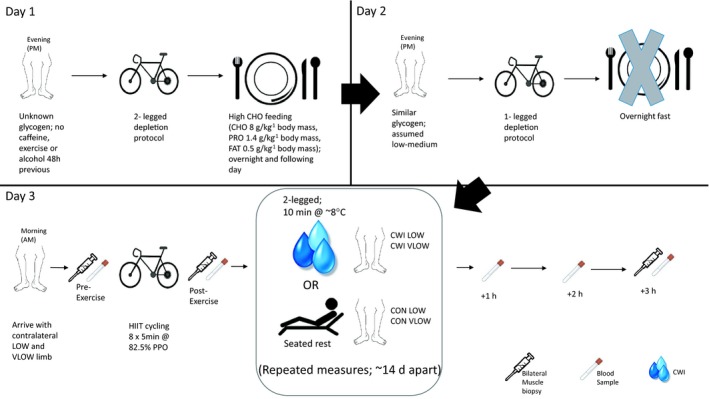
Overview of the experimental protocol used in each trial. HIIT, High‐intensity intermittent exercise; CHO, carbohydrate; PRO, protein; CWI, cold water immersion condition; PPO, peak power output; CON, control condition; LOW, low CHO limb; VLOW, very low CHO limb.

In a repeated‐measure, randomized crossover design, with at least 14 days between trials, participants reported to the laboratory a total of nine times, where the first three were familiarization sessions. In order to establish a research design that allowed the investigation of four separate conditions from two visits participants underwent a single‐leg depletion protocol and bi‐lateral muscle biopsies with and without post‐exercise CWI to give the following conditions: Low glycogen control (CON LOW), Very Low glycogen control (CON VLOW), Low glycogen CWI (CWI LOW) and Very Low glycogen CWI (CWI VLOW) (See Fig. 1).

### Two‐legged glycogen depletion, Visit 1

Participants arrived at the laboratory 40 h prior to the experimental trial at 1600 h and undertook a 5‐min warm‐up at 100 W. From here, participants performed an intermittent cycling protocol aimed to deplete both limbs of muscle glycogen. A two‐legged glycogen depleting cycling protocol consisting of 2 min at 90% PPO, followed immediately by a 2‐min recovery period at 50% PPO. Participants repeated this work to rest ratio until 2‐min cycling at 90% PPO could not be maintained, determined as an inability to maintain a cadence of 70 rev min^−1^. At this point, exercise intensity was lowered to 80% PPO, with the same work to rest ratio. When participants could no longer maintain this intensity, it was lowered to 70% and then finally to 60% PPO with the same work to rest ratio. When the participants were unable to cycle for 2 min at 60% PPO, the exercise protocol was terminated. This intermittent pattern of exercise has previously been shown to evoke glycogen depletion in both type I and type II fibres (Kuipers et al. 1987). After the completion of the two‐legged glycogen depletion protocol, participants were provided with a high CHO diet for the next ~22 h (CHO 8 g·kg^−1^ body mass, protein (PRO) 1.4 g·kg^−1^ body mass, Fat 0.5 g·kg^−1^ body mass). Feeding began immediately after the cessation of exercise for 4× hourly intervals that evening. Participants were also provided with breakfast for the following morning and returned to the laboratory post‐breakfast to collect food for the rest of the day (~8 g CHO·kg^−1^ body mass). The purpose of this initial glycogen depletion protocol with high CHO refeed was to ensure participants had a similar bi‐lateral concentration of muscle glycogen prior to commencing the second evening depletion.

### Single‐leg glycogen depletion, Visit 2

Approximately 15 h prior to the experimental trial, participants attended the laboratory for a single‐leg glycogen depletion protocol to deplete their dominant leg only. Glycogen depletion of the dominant leg was undertaken as to ensure similar muscle recruitment patterns and therefore glycogen depletion between trials. Single‐leg glycogen depletion involved 20 min continuous single‐leg cycling at 75% PPO, followed by intermittent cycling at a work to rest ratio of 90s:90s. Intermittent cycling began at 90% PPO decreasing in 5% decrements when such a workload could not be maintained for 5 s consecutively. Exercise ceased when 55% PPO could not be maintained for 5 s consecutively. Immediately following this, the participants completed an all‐out one‐legged cycling bout at 85% PPO before going on to 30 min of 2‐arm cycling at 50W in an attempt to decrease liver glycogen levels and therefore diminish the potential for muscle glycogen resynthesis (Pilegaard et al. 2002). Participants then underwent an overnight fast before returning to the laboratory the next day.

### Experimental trial, Visit 3

Upon arrival at the laboratory participants were fitted with a heart‐rate belt (Polar RS400, Kempele, Finland), skin and rectal temperature probes (MHF‐18050‐A and MRV‐55044‐A, Ellab, Rodovre, Denmark) and legs were marked for subsequent insertion of muscle temperature needles. Following 10‐minutes in a supine position baseline measures of HR, temperature and oxygen uptake ($\dot{V}O_{2}$; Oxycon Pro, Jaeger, Wuerzberg, Germany) were assessed. Resting venous blood samples were drawn from a superficial vein in the anti‐cubital crease of the forearm using venepuncture cannulation (BD Nexiva Closed IV Catheter 22G Blue, Becton Dickinson, Oxford, UK). Resting muscle temperature was assessed using a needle thermistor (13050; Ellab, Rodovre, Denmark) before resting bi‐lateral muscle biopsies were sampled from the vastus lateralis (~30–50 mg wet wt). Immediately after the resting biopsy participants completed a high‐intensity intermittent cycling protocol, consisting of 8 × 5 min bouts at 82.5% PPO separated by 1 min rest followed by either two‐legged CWI (CWI: 10 min at 7.96 ± 1.05°C) or a control condition (CON; seated rest). From here, participants recovered in a semi‐reclined position under normal laboratory temperatures until 3‐hours post‐exercise. Measures of heart rate, skin temperature (thigh and calf) and rectal temperature were recorded throughout the exercise and recovery periods. Oxygen uptake was measured during the final minute of each high‐intensity bout of exercise, during immersion and immediately post‐immersion to assess for shivering thermogenesis and again at 1, 2, and 3 h post‐exercise.

Laboratory temperatures remained stable throughout (~21°C) and at no point where participants allowed to rub themselves dry or shower (changing into dry clothes after immersion was allowed). Muscle temperature was assessed post‐exercise, 1, 2, and 3 h post‐exercise, whilst venous blood samples were also drawn at these times. Further bi‐lateral muscle biopsies occurred immediately after exercise and 3 h post‐exercise in line with previous research (Allan et al. 2017; Bartlett et al. 2012; Ihsan et al. 2014; Impey et al. 2016; Joo et al. 2016). Biopsies were obtained from both limbs at all time points to allow for comparison between low (LOW) and very low (VLOW) glycogen limbs in both CON and CWI trials. All incisions were individually anaesthetized, separated distally by 2–3 cm and included four passes of muscle tissue per biopsy.

### Blood analysis

All samples were analyzed in duplicate. Samples were analyzed for serum glucose, lactate, NEFA, and glycerol concentration using commercially available kits (Randox Laboratories, Antrim, UK). Plasma metanephrine and Normetanephrine concentrations were measured using liquid chromatography tandem mass spectrometry as previously described (Peaston et al. 2010). Serum samples were also analyzed for insulin using a solid phase enzyme‐linked immunosorbent assay (ELISA, KAQ1251, Life Technologies, UK), according to the manufacturer's instructions.

### Muscle glycogen

Muscle glycogen concentration was determined according to the method described by Van Loon et al. (2000). Approximately 2–3 mg of freeze‐dried sample was dissected free of all visible non‐muscle tissue and subsequently hydrolyzed by incubation in 500 μl of 1 mol L^−1^ HCl for 3–4 h at 100°C. After cooling to room temperature, samples were neutralized by the addition of 250 μl 0.12 mol L^−1^ KOH saturated with KCl. Following centrifugation, 150 μL of the supernatant was analyzed in duplicate for glucose concentration according to the hexokinase method using a commercially available kit (GLUC‐HLK, Randox Laboratories, Antrim, UK). Glycogen concentration is expressed as mmol·kg^−1^ dw and intra assay coefficients of variation was < 5%.

### RNA isolation and extraction

Two hundred microliter of chloroform was added per 1 mL TRIzol reagent used during homogenization and shaken vigorously by hand for 15 s before being incubated at room temperature for 3 min. Samples were then centrifuged at 12,000 g for 15 min at 4°C. After centrifugation, the samples were separated into their red phenol, middle interphase, and upper aqueous phase. The upper aqueous phase was carefully removed into a clean, labeled RNA/DNA free Eppendorf, ensuring the middle interphase was not disturbed, and mixed with 500 μL isopropanol (per 1 mL TRIzol). After vortexing for 15 s, the sample was incubated at room temperature for 10 min before further centrifugation (12,000 g for 10 min at 4°C). The resulting supernatant was removed and the remaining RNA pellet washed in 1 mL ice‐cooled 75% ethanol (per 1 mL TRIzol), vortexed briefly before centrifugation at 7500 g for 8 min at 4°C. The ethanol was subsequently removed and the RNA pellet allowed to air dry before re‐suspension in 30 μL RNA storage solution (Invitrogen, UK). Samples were incubated in a block heater at 50°C for 10 min to assist with re‐suspension before proceeding to measurement. RNA concentration and purity were assessed by UV spectroscopy at optical densities of 260 and 280 nm with the use of a Nanodrop 2000 (Thermo Fisher Scientific, UK). A target of A260/A280 ratio was set at 2.0. 70 ng RNA was used for each polymerase chain reaction (PCR) reaction.

### Primer design

Primer sequences (Table 1) were identified using Gene (NCBI, http://www.ncbi.nlm.nih.gov.gene) and designed using Primer‐BLAST (NCBI, http://www.ncbi.nlm.nih.gov/tools/primer-blast). Sequence homology searches ensured specificity. The primers were ideally designed to yield products spanning exon–exon boundaries to prevent any amplification of gDNA. Three or more GC bases in the last five bases at the 3’ end of the primer was avoided. Secondary structure interactions (hairpins, self‐dimer, and cross dimer) within the primer were avoided. All primers were between 16 and 25 bp, and amplified a product of between 67 and 212 bp. Primers were purchased from Sigma (Suffolk, UK).

**Table 1:** Primer sequences used for real‐time polymerase chain reaction

Gene	Forward primer	Reverse primer	Product length (base pairs)
**GAPDH** NM_002046.5	AAGACCTTGGGCTGGGACTG	TGGCTCGGCTGGCGAC	168
**PGC‐1alpha** NM_013261.3	TGCTAAACGACTCCGAGAA	TGCAAAGTTCCCTCTCTGCT	67
**p53** NM_000546.5	ACCTATGGAAACTACTTCCTGAAA	CTGGCATTCTGGGAGCTTCA	141
**SIRT1** NM_012238.4	CGGAAACAATACCTCCACCT	CACATGAAACAGACACCCCA	186
**COXIV** NM_001861.4	CGAGCAATTTCCACCTCTGT	GGTCACGCCGATCCATATAA	94
**CS** NM_004077.2	CCTGCCTAATGACCCCATGTT	CATAATACTGGAGCAGCACCCC	137
**TFAM** NM_003201.2	TGGCAAGTTGTCCAAAGAAACCTGT	GTTCCCTCCAACGCTGGGCA	135
**NRF2** NM_002040.3	AAATTGAGATTGATGGAACAGAGAA	TATGGCCTGGCTTACACATTCA	95
**ERRα** NM_004451.4	TGCCAATTCAGACTCTGTGC	CCAGCTTCACCCCATAGAAA	212
**GLUT4** NM_001042.2	TCTCCAACTGGACGAGCAAC	CAGCAGGAGGACCGCAAATA	101

Glyceraldehyde 3‐phosphate dehydrogenase –GAPDH; Peroxisome Proliferator‐activated receptor gamma coactivator 1‐alpha – PGC‐1α; Tumour suppressor protein 53‐ p53; Sirtuin 1 – SIRT1; Cytochrome C oxidase subunit 4 – COXIV; Citrate synthase – CS; Mitochondrial transcription factor A – TFAM; Nuclear respiratory factor 2 – NRF2; Estrogen‐related receptor alpha – ERRα; Glucose transporter type 4 – GLUT4.

### Gene expression analysis by real time‐quantitative reverse transcriptase polymerase chain reaction rt‐qRT‐PCR

rt‐qRT‐PCR amplifications were performed using QuantiFast^TM^SYBR^®^ Green RT‐PCR one‐step kit on a Rotor‐gene 3000Q (Qiagen, Crawley, UK) supported by rotor‐gene software (Hercules, CA). rt‐qRT‐PCR was performed as follows: hold 50°C for 10 min (reverse transcription/cDNA synthesis), 95°C for 5 min (transcriptase inactivation and initial denaturation step) and PCR steps of 40 cycles; 95°C for 10 s (denaturation) and 60°C for 30 s (annealing and extension). Upon completion, dissociation/melting curve analysis were performed to reveal and exclude non‐specific amplification or primer–dimer issues (all melt analysis in this study presented single reproducible peaks for each target gene suggesting amplification of a single product). Following initial screening of suitable reference/housekeeping genes, Glyceraldehyde 3‐phosphate dehydrogenase (GAPDH) showed the most stable Ct values across all RT‐PCR runs, participants and regardless of experimental condition (27.02 ± 1.96 CT; 7% Co‐efficient of variation) and was selected as the reference gene in all RT‐PCR assays. The relative gene expression levels were calculated using the comparative Ct (^ΔΔCt^) equation (Schmittgen and Livak 2008) where the relative expression was calculated as 2^−ΔΔCt^ and where Ct represents the threshold cycle. mRNA expression for all target genes was calculated relative to the reference gene (GAPDH; participants own reference, not pooled) within same subject and condition and to a calibrator of pre‐exercise. The average PCR efficiency was 91.25% and variation for all genes (including the reference gene) was <6.3%.

### Statistical analysis

All data are presented as mean ± SD. Baseline data, distance cycled, exercise HR and RPE were compared between conditions using a Paired Samples T‐test. A two‐way (4 conditions × time) within participant's general linear model for condition (CON LOW, CON VLOW, CWI LOW, CWI VLOW) and time was used to evaluate muscle glycogen, thigh skin temperature, muscle temperature, and gene expression. A two‐way (2 conditions × time) within‐participants general linear model was used to evaluate all blood measures (glucose, lactate, NEFA, glycerol, insulin, normetanephrine, metanephrine), rectal temperature, subjective and physiological responses (HR, RPE, shivering, $\dot{V}O_{2}$). The main effects for condition and time were followed up using planned LSD multiple comparisons. The ES magnitude was classified as trivial (<0.2), small (>0.2–0.6), moderate (>0.6–1.2), large (>1.2–2.0), and very large (>2.0–4.0) (Hopkins et al. 2009). The α level for evaluation of statistical significance was set at *P* < 0.05.

## Results

### Day 1 and 2: Glycogen depletion exercise protocols

In the two‐legged glycogen depletion protocol undertaken on the evening of Day 1, no difference was observed between conditions for distance cycled (CON 67.1 ± 18.1 km, CWI 67.3 ± 6.1 km, *P *=* *0.394, ES 0.01 Trivial), time to depletion (CON 134 ± 30 min, CWI 130 ± 12 min, *P *=* *0.669, ES 0.19 trivial) or number of intervals completed (8 ± 2 per stage for both CON and CWI, *P *=* *0.669, ES 0.20 Small). In the single‐leg glycogen depletion protocol undertaken on the evening of Day 2, no significant difference was present between conditions for distance cycled in the 20‐minute steady‐state period (CON 9.5 ± 1.2 km, CWI 8.5 ± 0.9 km, *P *=* *0.194, ES 1.09 Moderate), number of subsequent high intensity bouts completed (CON 6.8 ± 2.0 per stage, CWI 6.4 ± 2.0 per stage, *P *=* *0.235, 0.18 Trivial) and the distance cycled during the intervals (CON 47.5 ± 8.8 km, CWI 47.9 ± 7.3 km, *P *=* *0.587, ES 0.06 Trivial). Moreover, no differences were observed between conditions for time (CON 40.7 ± 9.0 s, CWI 37.5 ± 8.4 s) and distance (CON 0.6 ± 0.2 km, CWI 0.4 ± 0.1 km) completed in the all‐out exhaustive one‐leg cycle (*P *=* *0.197 ES 0.38 Small and 0.094 ES 0.94 Moderate, respectively).

### Day 3: Main experimental trial

#### Physiological responses to exercise

Distance cycled during the two‐legged high‐intensity intermittent cycling protocol (CON 26.8 ± 2.8 km, CWI 26.7 ± 3.4 km; *P *=* *0.946, ES 0.01 Trivial), heart rate (*P *=* *0.992, ES 0.004 Trivial), $\dot{V}O_{2}$ (mL·kg^−1^·min^−1^; *P *=* *0.602, ES 0.24 Small), and RPE (*P *=* *0.849, ES 0.07 Trivial) were similar between CON and CWI trials (data not shown). Mean HR during the final minute of exercise was 179 ± 7 beats·min^−1^ in CON and 177 ± 9 beats·min^−1^ in CWI (*P *>* *0.05), equating to ~80% HR max. The RPE in the final exercise bout was 20 AU and 19 AU in the CON and CWI trials, respectively. Such data highlight that the distance cycled and both whole body physiological/perceptual responses were comparable between the control and CWI trials.

#### Muscle glycogen concentration

**Figure 2:**
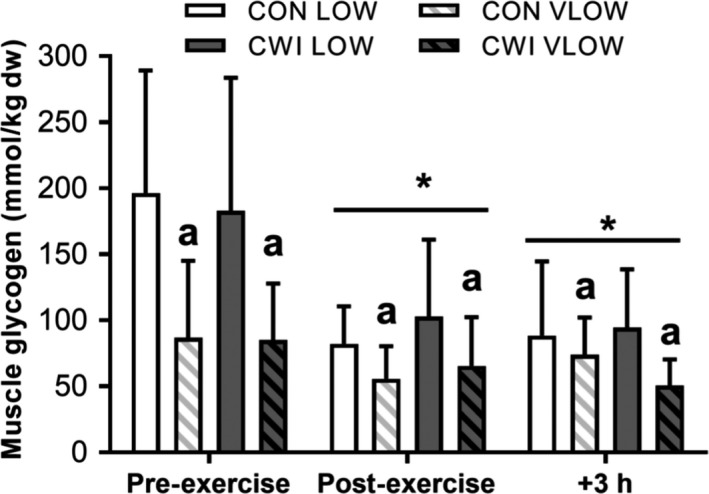
Skeletal muscle glycogen content immediately pre‐ and post‐exercise and after 3 h of recovery. Biopsies were obtained from both limbs in each condition (CON or CWI) with limbs starting the day being low (LOW) or very low (VLOW) in glycogen stores. A main effect for time (*P *= 0.001) and condition (*P *= 0.008) was observed. No interaction effects were present (*P *> 0.05). *Significantly different from PRE. a Significantly lower than contralateral LOW limb (*P *< 0.05). Data are mean ± SD.

Muscle glycogen concentrations were lower in the VLOW limbs compared to the LOW limbs in both the control (CON LOW vs. CON VLOW: *P *=* *0.017, ES 1.04 Moderate) and CWI trials (CWI LOW vs. CWI VLOW: *P *=* *0.001, ES 1.18 Moderate; Fig. 2). In contrast, the concentration of muscle glycogen was similar between the respective LOW (CON LOW vs. CWI LOW: *P *=* *0.819, ES 0.07 Trivial) and VLOW conditions (CON VLOW vs. CWI VLOW: *P *=* *0.751, ES 0.14 Trivial; Fig. 2). Muscle glycogen decreased immediately following exercise (*P *=* *0.001, ES 1.11 Moderate) and at 3 h post‐exercise (*P *=* *0.008, ES 1.10 Moderate) compared to pre‐exercise values, the magnitude of which did not differ between trials (*P *=* *0.116). Such data confirm that the intended aim of obtaining both LOW (i.e., <300 mmol·kg^−1^ dw) and VLOW (i.e., <150 mmol·kg^−1^ dw) pre‐exercise muscle glycogen concentrations was achieved in both the control and CWI trials.

#### Physiological and shivering responses to CWI versus control conditions

Heart rate was similar between conditions (*P *=* *0.584, ES 0.18 Trivial) during the immersion and recovery period (see Table 2). The change in HR over time was also similar between conditions (*P *=* *0.137), declining during the post‐immersion period in both conditions. During the same period, oxygen uptake was greater in CWI versus CON (*P *=* *0.014, ES 1.83 Large). The change in $\dot{V}O_{2}$ over time was also different between conditions (*P *=* *0.045) with increases in $\dot{V}O_{2}$ occurring during the initial 2 min of immersion. Following CWI, $\dot{V}O_{2}$ decreased below pre‐ immersion values and remained lower throughout the 3 h recovery period (ES > 0.58 Small) (*P *<* *0.05). Subjective ratings of shivering were greater in CWI (*P *=* *0.052, ES 0.94 Moderate). The change in subjective ratings of shivering over time also tended to be greater in CWI with values increasing during immersion and the 10‐min period immediately post‐immersion (*P* = 0.089) (Table 2).

**Table 2:** Heart rate (*n *=* *9), oxygen uptake (*n *=* *8), and subjective shivering measures (*n = *9) during immersion and the post‐immersion period (mean ± SD)

		PreIm	Immersion					Post‐Immersion							
			2 min	4 min	6 min	8 min	10 min	2 min	4 min	6 min	8 min	10 min	1 h	2 h	3 h
HR (beats·min^−1^)	CON	84 ± 5	81 ± 4	82 ± 2	80 ± 3	78 ± 5	82 ± 7	84 ± 5	83 ± 5	80 ± 6	82 ± 5	80 ± 4	79 ± 5	76 ± 8	74 ± 7
	CWI	95 ± 19	110 ± 20	94 ± 6	85 ± 12	81 ± 4	89 ± 18	79 ± 10	76 ± 9	74 ± 8	76 ± 11	80 ± 11	78 ± 9	69 ± 9	73 ± 12
$\dot{V}O^{2}$ (ml·kg^−1^·min^−1^)	CON	5.4 ± 0.8	5.0 ± 0.7	4.7 ± 0.6	4.6 ± 0.7	4.4 ± 0.6	4.6 ± 0.6	4.7 ± 0.6	4.5* ± 0.7	4.3* ± 0.5	4.9* ± 0.8	4.7* ± 0.7	4.6* ± 0.6	4.6* ± 0.8	4.2* ± 0.7
	CWI	8.5 ± 2.0	9.7 ± 2.1	9.0 ± 1.6	8.8 ± 2.1	8.4 ± 2.1	8.8 ± 2.6	6.8 ± 1.8	6.9* ± 1.9	6.2* ± 1.3	6.4* ± 1.7	6.3* ± 1.6	6.0* ± 1.5	5.4* ± 1.9	5.6* ± 1.5
		Pre Im			5 min		10 min	2 min	4 min	6 min	8 min	10 min	1 h	2 h	3 h
Subjective Shivering	CON	1 ± 0			1 ± 0		1 ± 0	1 ± 0	1 ± 0	1 ± 0	1 ± 0	1 ± 0	1 ± 0	1 ± 0	1 ± 0
(AU)	CWI	1 ± 0			2 ± 1		2 ± 1	2 ± 1	2 ± 1	2 ± 1	2 ± 1	2 ± 1	1 ± 0	1 ± 0	1 ± 0

PreIm, Pre Immersion; AU, arbitrary units.

Values are mean ± SD. A main effect for condition and time along with a significant interaction between condition and time was found for $\dot{V}O_{2}$ (*P* < 0.05).

* Significant difference from pre‐immersion (*P *<* *0.05).

#### Thermoregulatory responses to CWI versus control conditions

**Figure 3:**
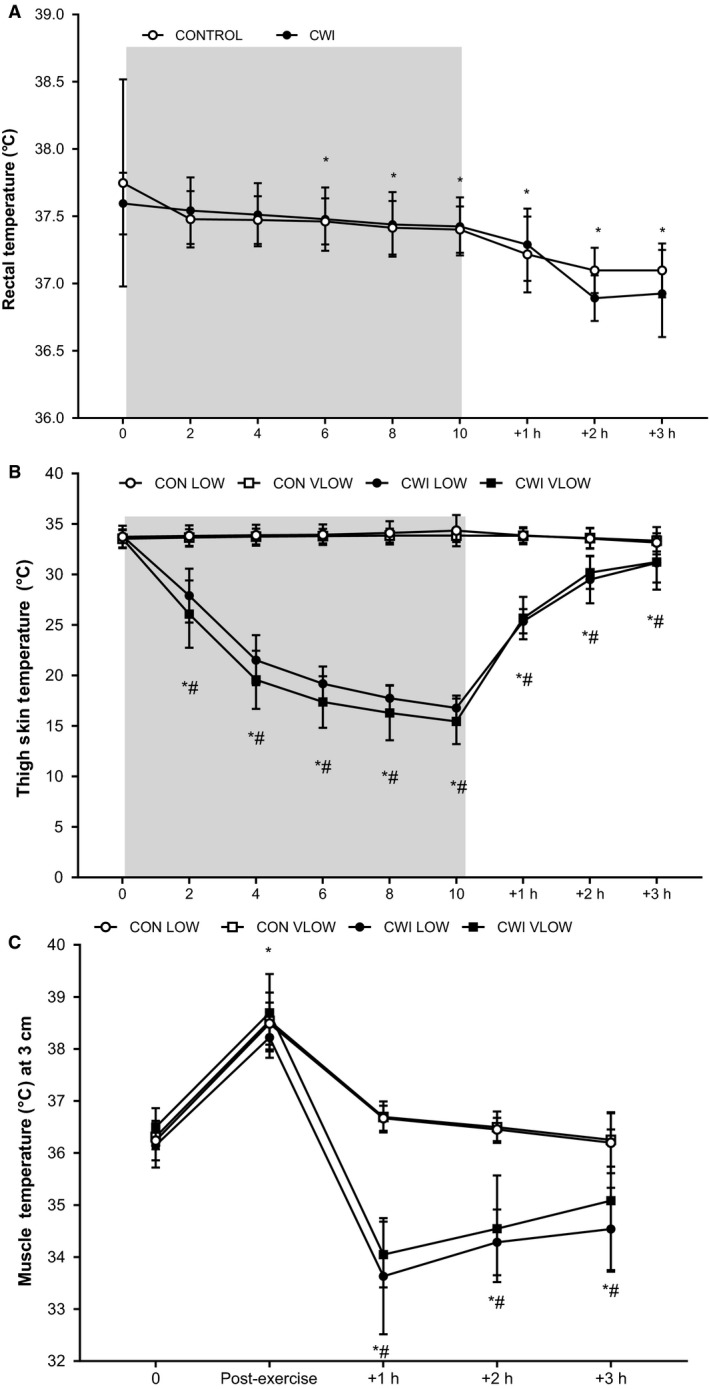
Rectal temperature (°C) (a), thigh skin temperature (°C) (b), and deep muscle temperature (3 cm; °C) (c) during immersion and the 3 h post‐exercise period. *Significantly different from pre‐. ^#^Significantly different from CON (*n* = 9 skin, *n* = 8 rectal, muscle; mean ± SD). Shaded area represents CWI.

Rectal temperature (Trec) was similar between conditions (*P *=* *0.887, ES 0.02 Trivial) during exercise and post‐exercise recovery (see Fig. 3A). During the immersion and recovery period, Trec decreased from the fifth minute of immersion until 3 h post‐exercise (*P *<* *0.001). Thigh skin temperature (Tthigh) was lower during immersion and the post‐immersion period in CWI vs. CON conditions (*P *<* *0.001, ES 2.48 Very Large). The change in Tthigh over time was also different between conditions, with Tthigh continually decreasing during cooling in CWI conditions, remaining lower than pre‐immersion values at 3 h post exercise (*P *=* *0.001, see Fig. 3B). Muscle temperature (Tmus) was lower following immersion (*P *<* *0.001) in the CWI limbs (*P *<* *0.001, ES 0.90 Moderate). The change in Tmus over time was also different between conditions. Muscle temperature declined to a large extent immediately after immersion in the CWI limb, followed by a further gradual reduction during the remaining 3 h post‐exercise period (*P *<* *0.001, ES 1.0 Moderate, see Fig. 3C).

**Table 3:** Plasma‐derived metabolic and hormonal markers measured Pre‐Exercise, Post‐Exercise, +1 h, +2 h, +3 h following the cessation of exercise (*n *=* *9 mean ± SD)

		Pre‐exercise	Post‐exercise	+1 h	+2 h	+3 h
Glucose (mmol·L^−1^)	CON	5.50 ± 0.50	6.15 ± 0.37*	5.28 ± 0.32*	5.20 ± 0.52*	5.29 ± 0.36*
	CWI	5.77 ± 0.57	6.06 ± 0.89*	5.30 ± 0.85*	5.25 ± 0.85*	5.32 ± 0.76*
Lactate (mmol·L^−1^)	CON	1.41 ± 0.40	8.23 ± 3.97*	1.86 ± 0.94	1.32 ± 0.44	1.36 ± 0.47
	CWI	1.68 ± 0.69	7.47 ± 3.42*	2.78 ± 1.55	1.75 ± 0.65	1.44 ± 0.38
NEFA (mmol·L^−1^)	CON	0.60 ± 0.24	0.83 ± 0.26	1.39 ± 0.23*	1.44 ± 0.35*	1.49 ± 0.43*
	CWI	0.65 ± 0.24	0.87 ± 0.38	1.64 ± 0.29*	1.39 ± 0.33*	1.50 ± 0.29*
Glycerol (μmol·L^−1^)	CON	44.94 ± 23.70	290.22 ± 99.92*	120.78 ± 51.52*	114.63 ± 50.00*	102.36 ± 45.79*
	CWI	41.11 ± 22.02	273.81 ± 52.52*	155.36 ± 38.32*	116.00 ± 52.46*	97.21 ± 30.75*
Insulin (U·mL^−1^)	CON	12.88 ± 6.86	11.55 ± 3.06	13.50 ± 4.08	12.55 ± 4.68	12.79 ± 6.47
	CWI	14.04 ± 5.02	10.32 ± 4.02	11.61 ± 5.67	11.57 ± 3.61	6.53 ± 1.17
Normetanephrine (pmol·L^−1^)	CON#	699.81 ± 197.68	1728.39 ± 481.09*	914.39 ± 275.69*	738.82 ± 247.92*	634.93 ± 204.63
	CWI#	553.09 ± 237.16	1883.33 ± 655.17*	1128.64 ± 531.83*	1033.08 ± 461.74*	919.57 ± 371.43
Metanephrine (pmol·L^−1^)	CON	263.94 ± 133.08	535.48 ± 145.29*	305.63 ± 98.15*	272.43 ± 49.25	232.32 ± 72.05
	CWI	245.67 ± 80.04	506.73 ± 149.39*	321.30 ± 88.33*	258.46 ± 75.54	268.76 ± 70.56

* Significantly different from Pre‐exercise (*P *<* *0.05).

# Main interaction effect present (*P = *0.026).

#### Circulating plasma metabolites and catecholamines

There was no significant difference in plasma glucose, lactate, NEFA, insulin, and glycerol concentrations between conditions (*P *>* *0.05) (Table 3). The change in these parameters over time was also similar between conditions (*P* > 0.05). Exercise‐induced significant increases in glucose, lactate, glycerol, and NEFA (*P* < 0.05). Metanephrine concentrations were similar between conditions (*P *=* *0.955, ES 0.02 Trivial, Table 3). The change in metanephrine over time was also similar between conditions (*P *=* *0.438). Metanephrine concentration was increased post‐exercise (*P *<* *0.001, ES 2.10 Very Large) and remained above baseline at 1 h post‐exercise (*P *=* *0.02, ES 0.59 Small). Normetanephrine concentrations were similar between conditions (*P *=* *0.130, ES 0.14 Trivial, Table 3). The change in normetanephrine over time was different between conditions, with normetanephrine concentrations decreasing to a greater extent in CON during the 3 h post‐exercise period (*P *=* *0.026, ES 1.60 Large). Normetanephrine concentration increased post‐exercise (*P *=* *0.002, ES 1.50 Large) and remained above baseline in CWI conditions until 2 h post‐exercise (*P *<* *0.05; *P *=* *0.058).

#### Skeletal muscle mRNA responses

**Figure 4:**
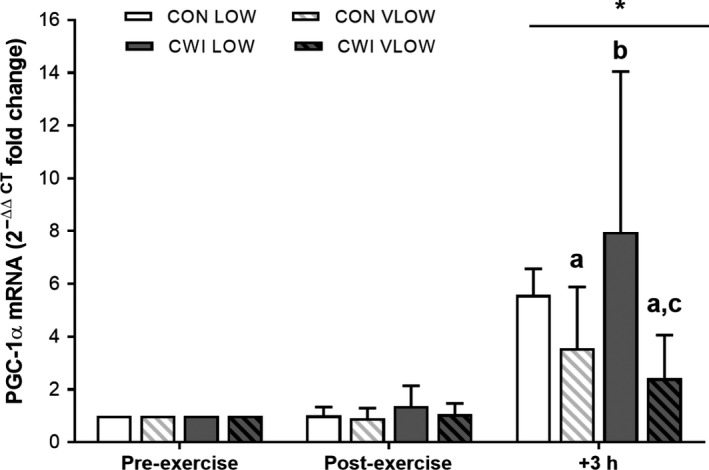
PGC‐1α mRNA 2^−ΔΔCT^ fold change in expression with the calibrator as preexercise and the reference gene as GAPDH (see methods for details). Values are mean ± SD. A time × condition interaction effect was observed (*P *= 0.034). * significantly greater than Pre‐ and Post‐Exercise (*P < *0.001). ^a^significantly less than CON LOW (*P *< 0.05), ^b^significantly greater than CON VLOW (*P *= 0.05), ^c^significantly less than CWI LOW (*P *= 0.019).

**Figure 5:**
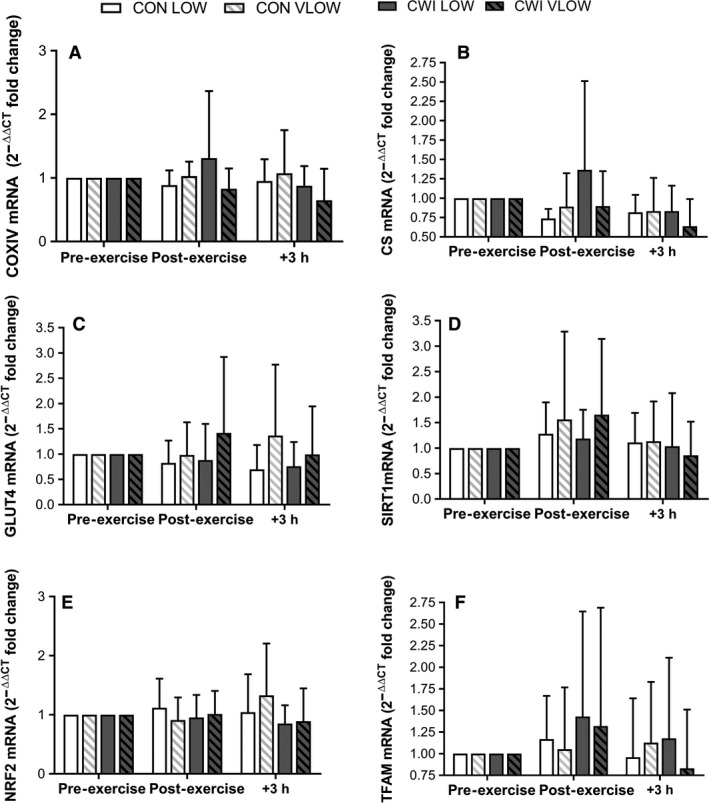
mRNA 2^−ΔΔCT^ fold change in expression with the calibrator as pre‐exercise and the reference gene as GAPDH (see methods for details). Values are mean ± SD.

Exercise increased PGC‐1α mRNA at 3 h post‐exercise in all conditions (*P *<* *0.001; ES 1.99 Large) (Fig. 4). At 3 h post‐exercise, PGC‐1α mRNA expression was attenuated in the VLOW limbs compared with the LOW limbs in both the CON (CON VLOW vs. CON LOW: *P *=* *0.023, ES 1.22 Large) and CWI conditions (CWI VLOW vs. CWI LOW: *P *=* *0.019, ES 1.43 Large; *P *=* *0.039). This reflected the greater change in expression in the LOW limbs in both CON and CWI conditions between post‐exercise and 3 h post‐exercise time points (*P *=* *0.034). There was no significant difference in PGC‐1α mRNA expression between the CWI LOW limb and CON LOW limb at 3 h post‐exercise (*P *=* *0.281, ES 0.67 Moderate) (Fig. 4). In contrast to PGC‐1α, the expression of COXIV, CS, TFam, SIRT1, NRF2, and GLUT4 mRNA (Fig. 5) did not change in response to exercise or CWI (*P* > 0.05).

## Discussion

The rationale for the present study was based on previous observations that both post‐exercise CWI (Allan et al. 2017; Ihsan et al. 2014, 2015; Joo et al. 2016) and reduced muscle glycogen availability (Bartlett et al. 2013; Impey et al. 2016) independently augment the exercise‐induced mRNA expression of the master regulator of mitochondrial biogenesis, PGC‐1α. Accordingly, it is tempting to speculate that the application of both stressors simultaneously amplifies the adaptive responses of skeletal muscle to exercise, when compared with either intervention alone. In contrast, given recent data highlighting the role of local metabolic stress in modulating acute exercise‐induced cell signaling pathways (Fiorenza et al. 2018), we hypothesized that the application of CWI (i.e., a systemic mediated stress) induces negligible regulatory effects on muscles that have already been subjected to the extreme local metabolic challenge of both high‐intensity exercise and low muscle glycogen availability. Confirming our hypothesis, we demonstrate that the application of post‐exercise CWI does not enhance the exercise‐induced expression of PGC‐1α mRNA in muscles that completed an acute high‐intensity cycling protocol with low (i.e., <300 mmol·kg^−1^ dw) or very low (i.e., <150 mmol·kg^−1^ dw) pre‐exercise muscle glycogen concentrations. From a practical perspective, our data suggest that the application of post‐exercise CWI as a strategic training aid for greater PGC‐1α gene expression is more likely to have beneficial effects when utilized after those high‐intensity training sessions that have not induced near maximal glycogen depletion.

In our previous study (Allan et al. 2017), we utilized the same exercise protocol as that studied here (i.e., 8 × 5 min at 82.5% PPO) and demonstrated that post‐exercise CWI (10 min of single limb immersion at 8°C) augments PGC‐1α mRNA expression (9–12‐fold) in both the immersed and non‐immersed limbs when compared with biopsies obtained from an exercise only trial after which no post‐exercise CWI occurred (fivefold). In using that specific design, these data suggested that the effects of post‐exercise CWI previously observed by our laboratory (Joo et al. 2016) and others (Ihsan et al. 2014, 2015) is regulated systemically via β‐adrenergic activation of AMPK (Allan et al. 2017) and/or cAMP‐CREB‐PGC‐1α signaling (Akimoto et al. 2008), as opposed to local cooling effects per se. In the present study, we recruited a similar subject population to complete the same two‐legged exercise protocol studied previously but in conditions where each limb commenced exercise with low (<300 mmol·kg^−1^ dw) or very low (<150 mmol·kg^−1^ dw) muscle glycogen availability. Additionally, we also adopted a two‐legged post‐exercise CWI protocol as opposed to the single limb immersion protocol studied previously (Allan et al. 2017). Indeed, given that the magnitude of sympathetic discharge to skeletal muscle is influenced by both the size of the tissue area exposed to cooling (Seals 1990) as well as the magnitude of the cooling stimulus (Kregel et al. 1992), it is noteworthy that the two‐legged immersion protocol studied here elicited almost double the stress response as to that observed previously in response to single limb immersion (i.e., Normetanephrine ~919 vs. ~517 pmol·L^−1^ at 3 h post‐exercise). Nonetheless, despite the enhanced cooling stimulus and adrenergic response observed here, we observed no augmented effects of post‐exercise CWI on PGC‐1α mRNA expression in either the LOW or VLOW limbs. When taken together, it could be suggested that the systemic effects of post‐exercise CWI (i.e., β‐adrenergic activation of AMPK and/or cAMP‐CREB‐PGC‐1α) induces negligible effects on the regulation of PGC‐1α mRNA expression when the relevant upstream signaling cascade(s) has already been activated by the combination of high‐intensity exercise and low muscle glycogen availability (i.e., glycogen mediated AMPK‐PGC‐1α signaling).

Surprisingly, one of the most novel aspects of the present study was the finding that the magnitude of the exercise‐induced changes in PGC‐1α was reduced in the VLOW limbs compared with the LOW limbs, a finding that was evident in both the CWI and control trials. It is difficult to offer a definitive explanation for this finding but we suggest two related reasons. Firstly, given that Ca^2+^ release from the sarcoplasmic reticulum (SR) is significantly impaired in glycogen depleted fibres (i.e., <150 mmol·kg^−1^ dw) (Ørtenblad et al. 2011), it is possible that force production was lower in the VLOW limbs when compared with the LOW limbs. As such, subjects may have exhibited greater muscle fiber recruitment in the LOW limbs when compared with the VLOW limbs in order to induce a compensatory effect to maintain gross cadence and power output. In this way, a lower PGC‐1α response (as detected in whole muscle homogenates) in the VLOW limbs may simply be explained by lower absolute muscle fiber recruitment. Alternatively, the potential reduction in SR Ca^2+^ release within specific muscle fibers (Ørtenblad et al. 2011) may actually reduce Ca^2+^ mediated regulation of the cyclic AMP response element of the PGC‐1α promoter owing to reduced upstream signaling through p38 MAPK, CaMKII, and CREB (Wright et al. 2007). Unfortunately, we cannot currently offer definitive support for this hypothesis given that we did not quantify muscle fiber recruitment of the vastus lateralis muscles of both the LOW or VLOW limbs, nor did we measure the activation status of the aforementioned signaling proteins in either whole muscle homogenate or specific muscle fibers. Nonetheless, the suggestion that extremely low muscle glycogen availability may impair exercise‐induced cell signaling (as opposed to enhance signaling) lends support for the recently proposed muscle glycogen threshold hypothesis (Hearris et al. 2018) surmising that cell signaling processes are particularly responsive within a given range of absolute pre‐ to post‐exercise muscle glycogen concentrations (e.g., 300 to 100 mmol·kg^−1^ dw).

Importantly, the VLOW glycogen limbs underwent depleting exercise the night before the experimental day, while the LOW limbs did not. This may therefore have led to higher absolute pre‐exercise mRNA values in the VLOW versus LOW glycogen limbs, ultimately lowering the potential fold changes seen at 3 h post‐exercise when compared with pre‐values. It is important to highlight that gene expression in the present study was calibrated to the limbs own PRE value. As such, it is sensible to suggest that the lower values noted for PGC‐1α mRNA expression in the VLOW versus LOW limbs might be a factor of this calibration and higher basal levels in the VLOW limbs. Indeed, exploring further and calibrating all PGC‐1α mRNA data to CON LOW Pre‐Exercise the difference between LOW and VLOW conditions is no longer statistically significant (*P *>* *0.05). However, despite this, it still remains that post‐exercise CWI was unable to augment PGC‐1α mRNA above the exercise response, in the expected manner for LOW and VLOW conditions. This supports the earlier point that the muscle may have already been exposed to sufficient levels of metabolic stress via extremely low glycogen availability and therefore any additional stress from the cold is unable to augment PGC‐1α mRNA further. This further exploration is not surprising given the low availability of glycogen in both LOW and VLOW conditions.

In summary, we provide novel data demonstrating that the previously documented effects of post‐exercise CWI in modulating PGC‐1α mRNA expression in human skeletal muscle are not apparent when exercise is commenced with very low muscle glycogen availability. Such data suggest that any potential effect of systemically mediated regulation of PGC‐1α mRNA expression is negligible when muscles have already been exposed to sufficient local signaling events that arise during exercise. Additionally, the presence of extremely low muscle glycogen availability may actually impair exercise‐induced cell signaling processes. From a practical perspective, our data suggest that athletes are more likely to obtain beneficial PGC‐1α gene expression from post‐exercise CWI protocols when utilized after those high‐intensity interval‐training sessions that have not induced near maximal glycogen depletion.
